# Comment on ‘CD3+/CD4+ Cells Combined With Myosteatosis Predict the Prognosis in Patients Who Underwent Gastric Cancer Surgery’ by Du Et Al.

**DOI:** 10.1002/jcsm.13588

**Published:** 2024-09-28

**Authors:** Wenchu Dai, Jinlin Liu, Pan Zhao

**Affiliations:** ^1^ Department of Clinical Laboratory Xian No. 1 Hospital Xi'an China; ^2^ Department of Clinical Laboratory, South China Hospital, Medical School Shenzhen University Shenzhen China

Dear Editor,

Sir, we read Du's study [1] with great interest. In this study, they aimed to investigate the predictive capacity of lymphocyte subpopulations, sarcopenia and myosteatosis for clinical outcomes in patients who underwent gastric cancer surgery. Additionally, the prognostic significance of CD3+/CD4+ cells in conjunction with myosteatosis was explored. Based on their statistical analysis, they found that CD3+/CD4+ cells, CD4+/CD8+ ratio and CD19+ cells are indicative of clinical prognosis in gastric cancer surgery patients. Sarcopenia and myosteatosis are also associated with the patient's prognosis. Despite promising results, in this letter, we raise some statistical concerns about the results of this study.

Firstly, we noted that there were 38 variables in Table 2 or Table 3 for univariate and multivariate analysis for progression‐free survival or overall survival. Thus, according to the basic statistical rule demands that 1 covariate per 10 outcomes for the prediction analysis [2, 3, 4, 5], analysing 38 covariates demands at least 380 death gastric cancer patients in Du's study. In contrast, there were only a total of 190 gastric cancer patients, let alone fewer death patients (approximately <100 death patients) in this study. Thus, these logistic regression analysis models were too overfitted, the statistical result may not be accurate, and it needs another larger cohort to validate their risk factors's results.

Secondly, the author could reduce the number of covariates by making the comparison between the dead and survival groups, screening the reduced covariates and using these reduced variables to do the logistic regression analysis. It can get more reliable results.

Thirdly, did these gastric cancer patients receive chemotherapy in this study, while this chemotherapy could severely influence the results of haematological parameters? As staff worked in the clinical laboratory, we know that the haematological parameters were severely influenced by chemotherapy drugs. The same patient may have different results on the same day. Thus, the author should discuss the chemotherapy drug's effect on these covariates, such as the influence on the lymphocyte subsets and other haematological parameters. Without considering this influence, these gastric cancers may have different baselines, resulting in inaccurate risk factors results.

Finally, we show great respect for Du's outstanding study despite these comments.

## Conflicts of Interest

The authors declare no conflicts of interest.
